# A new grading system for female sexual dysfunction based on the female sexual function index in Egyptian women: a cross-sectional study

**DOI:** 10.4314/ahs.v21i2.44

**Published:** 2021-06

**Authors:** Sahar A Ismail, Nagwa E Abdel-Azim, Medhat A Saleh, Ahmed A Mohamed, Ali H Yosef, Ahmad M Abbas

**Affiliations:** 1 Department of Dermatology, Venereology and Andrology, Faculty of Medicine, Assiut University, Assiut, Egypt; 2 Department of Public Health and Community Medicine, Faculty of Medicine, Assiut University, Assiut, Egypt; 3 Department of Obstetrics & Gynecology, Faculty of Medicine, Assiut University, Assiut, Egypt

**Keywords:** Female sexual dysfunction, FSFI, grading, sexual function

## Abstract

**Objective:**

To provide a grading system that accurately reflects the grades of female sexual dysfunction (FSD) severity.

**Patients and methods:**

A cross-sectional study was conducted in Assiut University Hospital. It included 500 women who answered the Arabic version of the Female Sexual Function Index (FSFI) after getting their consent. A gradient of FSD severity was created, classifying FSD into five grades: severe, moderate, mild to moderate, mild, and no FSD.

**Results:**

According to our grading system, FSD was detected in 339 women (67.8 %); Mild FSD in 20.4%, mild to moderate in 41.6%, moderate in 15.3%, and severe in 22.7%. Mean scores of desire show a linear trend of reduction from 3.8 in mild to 3.36 in mild to moderate to 2.25 in moderate and markedly reduced to 2.1 in severe grade. This difference was highly statistically significant (p= 0.002). The same was reported in arousal, orgasm, and satisfaction domains, while in lubrication and pain domains, the difference was not statistically significant.

**Conclusion:**

In this study, our grading system was complementary to the FSFI. Moreover, it seems to be more practical and useful in grading the severity of FSD.

## Introduction

Female sexual dysfunction (FSD) is a prevalent health problem^1^. It is usually manifested by difficulties getting aroused, lubricated, or having an orgasm despite adequate stimulation^2^. In contrast to male sexual dysfunction, simple measures such as sexual activity cannot be used as an accurate indicator of dysfunction, as women may stay sexually active with their partners while suffering from some degree of sexual dysfunction^3^.

Many methods were developed to evaluate such problems in research and clinical settings. These include structured interviews, questionnaires, and detailed case histories. Questionnaires have become an easy first choice to screen individuals for FSD^4^. Among these questionnaires, Female Sexual Function Index (FSFI)^5^ is considered a “gold standard” measure to reflect female sexual function^6^. This questionnaire was shown to have both high test-retest reliability for each individual domain and a high degree of internal consistency. However, it classifies women into two categories only: normal and abnormal, with a cut-off score of 26.557. An Arabic version of FSFI was later validated by Anis et al.^8^, who calculated a total score of 28.1 as the cut-off score that defines sexual dysfunction in the Egyptian population.

The wide range of total FSFI scores denoting FSD (2-28.1), in addition to the high prevalence of FSD worldwide, highlighted the need for a grading system that can classify women with FSD into several severity levels. Moreover, the variability of FSD therapeutic options, the serious side effects of some of those therapies, and the lack of treatment guidelines for FSD are essential reasons for considering the need for a grading system.

## Patients and methods

This study aimed to provide a grading system for the FSFI that accurately reflects grades of FSD severity and to present data supporting its use as a diagnostically valid instrument in clinical settings worldwide.

A cross-sectional hospital-based study was conducted at the outpatient clinics of Dermatology, Venereology, and Andrology department, Assiut University Hospital from June 2016 to August 2017 after obtaining informed consent from all women who agreed to participate in the study and approving the proposal from the local ethical institutional review board. The target women were healthy, sexually active married Egyptian women aged 18 to 55 years who had visited the hospital for a routine check-up or mild dermatological illnesses. It also included women who had accompanied other patients. We excluded women with severe or chronic medical diseases, psychiatric illness, pregnancy, and lactation, and were not sexually active in the last six months.

We calculated the sample size according to the equation for the sample size of descriptive study design^9^; that is: N= {Z2(1-P)P}/D2 where: N: minimum sample size required, Z: standard normal variance=1.96 at 95% confidence interval, D: Absolute standard error that can be tolerated =0.05 and P: prevalence= 50% (based on previous studies in Egypt, Saudi Arabia, and Turkey)10-12. A sample size of at least 385 women was required to achieve the aim of the study. A total of 500 women were targeted to increase the statistical reliability of the study.

Women were interviewed in a private room with their names and addresses not recorded to ensure confidentiality. They were asked to fill out the Arabic version of the FSFI. This 19-item standardized questionnaire covers six domains: desire, arousal, lubrication, orgasm, satisfaction, and pain. It evaluates sexual function during the last month^8^. For each domain, a score was calculated, and the total score was obtained by adding the six domain scores. The total score range was 2 to 36. We developed a new grading system for determining the severity of FSD among the studied sample and named it (Sahar's grading system); this system tries to classify the total score obtained by FSDI into several grades, which can reflect its severity as shown in table (1).

**Table 1:** Female Sexual Function Index domain scores and full scale score

Domain	Questions	Score range	factor	Domain score (Response option x domain factor)					
				0–1	1	2	3	4	5
**Desire**	1,2	1–5	0.6	1.2	1.2	2.4	3.6	4.8	6
**Arousal**	3,4,5,6	0–5	0.3	0	1.2	2.4	3.6	4.8	6
**Lubrication**	7,8,9,10	0–5	0.3	0	1.2	2.4	3.6	4.8	6
**Orgasm**	11,12,13	0–5	0.4	0	1.2	2.4	3.6	4.8	6
**Satisfaction**	14,15,16	0 or (1)-5	0.4	0.8	1.2	2.4	3.6	4.8	6
**Pain**	17,18,19	0–5	0.4	0	1.2	2.4	3.6	4.8	6
**Total**				2	7.2	14.4	21.6	28.8	36

Our grading system depends on the answers to each question of FSFI. As each question has an ascending response score from 0/1 to 5, where 1 represents the lowest response score reflecting severe affection, and 5 represents the highest response score reflecting no affection. We concluded that the sum of the scores of all “1” answers would give the patient a total score reflecting severe FSD, and that of all “2” answers will give the patient a total score reflecting moderate FSD and so forth. FSD severity was then classified into the following five categories based on FSFI total scores; severe (2–7.2), moderate (7.3–14.4), mild to moderate (14.5–21.6), mild (21.7–28.1 “cutoff value”), and no FSD (28.2–36).

## Statistical analysis

All data were analyzed using SPSS software (Chicago, IL, USA) version 22. The results were expressed as mean±SD for quantitative data or frequencies (percentage) for qualitative data. We tested the different scores for normality by Shapiro-Wilks test for statistical analysis, and they were normally distributed. Chi-square test was used for comparison of qualitative data, while ANOVA test was used in the comparison of means in quantitative data. P values less than 0.05 were considered statistically significant.

## Results

A total of 583 healthy sexually active married females were approached, of whom 500 (85.8%) accepted to participate in the study. Their sociodemographic characteristics are shown in table (2). The age of the participants ranged from 17 – 55 years, with a mean ± SD of 32.94 ± 9.76 years. Most of them were in the age groups 30 to < 40 years and 20 to < 30 years (32.0% and 30.0 %, respectively). Regarding the level of education, 40.8% had a college education, (26.2%) had secondary education, and 14.0% were illiterate. Most of the participants were living in urban areas (57.4%), not working (59.2%), and circumcised (59.0%).

**Table 2:** Socio demographic characteristics of the study participants

Variable	Cases = 500 No. (%)
Age (in years): <20 20–<30 30–<40 40–<50 ≥50	48 (9.6) 150 (30.0) 160 (32.0) 118 (23.6) 24 (4.8)
Education: Illiterate: Primary: Secondary: University:	70 (14.0) 95 (19.0) 131 (26.2) 204 (40.8)
Work status: Unemployed (House wife): Employed:	296 (59.2) 204 (40.8)
Residence: Rural: Urban:	213 (42.6) 287 (57.4)
Age at marriage (years): <20 20–25 >25	129 (25.8) 218 (43.6) 153 (30.6)
Duration of marriage (years): <5 5–<10 10–15 >15	151 (30.2) 113 (22.6) 87 (17.4) 149(29.8)
Female genital mutilation: Yes No	345 (69.0) 155 (31.0)

**Figure 1:**
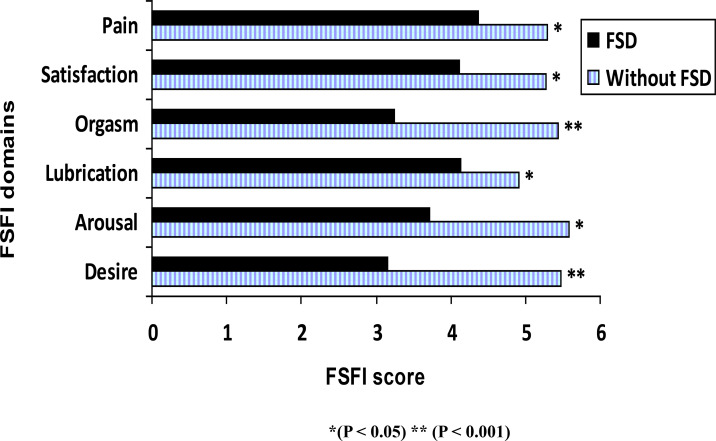
Mean score for each FSFI domain in women with and without sexual dysfunction.

Based on the total sexual function s6core, 339 women (67.8 %) had sexual dysfunction (Table 3). The mean FSFI score for women with sexual dysfunction was 16.73± 7.50 compared to 31.31±1.43 for women without sexual dysfunction, and this difference was statistically significant (P <0.001). Comparison of the individual domain scores revealed that women with sexual dysfunction had significantly lower scores for all domains compared with women without sexual dysfunction, with the lowest scores noted in the desire domain followed by orgasm and arousal domains (figure 1).

**Table 3:** Mean domains distribution in women with and without sexual dysfunction

Domains	Normal		Abnormal		Score Mean ± SD	P-value
	No.	%	No.	%		
**Desire**	161	32.2	339	67.8	3.62 ± 1.40	0.004*
**Arousal**	168	33.6	332	66.4	3.41 ± 1.97	0.008*
**Lubrication**	152	30.4	348	69.6	3.74 ± 2.00	0.024*
**Orgasm**	132	26.4	368	73.6	3.45 ± 1.91	0.002*
**Satisfaction**	155	31.0	345	69.0	4.01 ± 1.56	0.012*
**Pain**	52	10.4	448	89.6	3.18 ± 1.80	0.032*
**Total**	161	32.2	339	67.8	21.42 ± 9.24	0.001*

* Statistical significant difference

Table (4) shows the distribution of degree of severity of our grading system for FSD that was discussed in the methodology and revealed that; 69 women (20.4%) of the total sample were classified mild FSD, about two-fifths of the women (41.6%) were classified to have mild to moderate FSD, 15.3% of the women have moderate dysfunction, while 22.7 classified to have severe dysfunction.

**Table 4:** Distribution of grads of FSD according to Sahar's grading score

FSD grades	No. = 339	%
Mild FSD	69	20.4
Mild to moderate FSD	141	41.6
Moderate FSD	52	15.3
Severe FSD	77	22.7
Total	339	100.0

Table (5) demonstrates the relation between the degree of severity of FSD according to our grading system and mean score of the six domains of sexual function, and reveals a statistically significant difference in mean scores of 4 out of the six the domains (desire, arousal, orgasm, and satisfaction) while no statistically significant difference was observed in pain and lubrication. The mean score of desire shows a linear trend of decrease from 3.8 in mild to 3.36 in mild to moderate to 2.25 in moderate and markedly reduced to 2.1 in severe grade, and this difference was highly statistically significant (0.002). The same findings were reported in arousal, orgasm, and satisfaction domains, while in lubrication and pain domains, the difference was not statistically significant.

**Table 5:** Degree of severity of our score and its relation with each domain in the study participants

Domains	Sahar's classification of FSD					P-value
	Mild (69)	Mild to moderate (141)	Moderate (52)	Severe (77)	Total FSD (339)	
	Mean ± SD	Mean ± SD	Mean± SD	Mean± SD	Mean± SD	
**Desire**	3.80 ± 0.65	3.36 ± 0.91	2.25 ± 1.04	2.1 ± 1.19	3.01 ± 1.51	0.002*
**Arousal**	4.07 ± 0.48	2.91 ± 0.49	1.90 ± 0.50	0.16 ± 0.56	2.36 ± 1.49	0.001*
**Lubrication**	4.09 ± 0.68	3.51 ± 0.68	2.53 ± 0.68	0.00 ± 0.00	2.74 ± 1.67	0.07
**Orgasm**	4.23 ± 0.87	3.15 ± 0.80	2.30 ± 0.62	0.08 ± 0.23	2.54 ± 1.62	0.003*
**Satisfaction**	4.78 ± 0.85	3.20 ± 0.95	2.76 ± 0.12	2.27 ± 0.96	3.25 ± 1.28	0.009*
**Pain**	4.07 ± 0.82	3.91 ± 1.02	2.32 ± 1.29	0.02 ± 0.89	2.81 ± 1.86	0.3
**FSD**	25.27 ± 0.79	20.09 ± 2.02	14.04±1.65	4.73 ± 1.50	16.72 ± 7.50	0.001*

ANOVA test was used

* Statistical significant difference

## Discussion

FSD is a highly prevalent and usually underestimated problem. It is a multifactorial, progressive, and age-related problem. Few studies used validated instruments to determine this dysfunction in our community.

Our results reveal that 67.8% of participating women in our study were suffering from sexual dysfunction, which was a high prevalence. This was consistent with a previously reported prevalence of 75.3% by Anis et al.^8^. They used DSM-IV (Diagnostic and Statistical Manual of Mental Disorders, 4^th^ edition) in their study of 855 women from Lower Egypt^8^. In addition, it is consistent with the findings of two earlier studies in Egypt^13^, ^14^ that reported a prevalence of 68.9% and 76.9% of sexual dysfunction, respectively.

This high prevalence of FSD in our study, which was consistent with other previous studies in Egyptian women, addresses the need for studying the roots and determinants of this problem. On the other hand, a slightly lower prevalence of FSD (52.8 %) was reported by Ibrahim et al.^10^ in their study of 508 Egyptian women from the Suez district using FSFI10. The reason of lower prevalence in that study may be attributed to the fact that they used a cut-off value ≤ 26.55 total score, reported by Wiegel et al.^7^ in their analysis of a U.S. sample of women7 while we used the cut-off value ≤ 28.1 total score, reported by Anis et al.^8^ for Egyptian population8. This difference of the two cut-off values was attributed to a variety of cultural, educational, and ethnic differences between the two study populations, as women live in our study site (Upper Egypt) have low cultural and educational characteristics than those of Lower Egypt that may play a role in increasing sexual dysfunction among them.

Moreover, the use of different methods of determination, such as the ROC curve used by Anis et al.^8^ compared to the Classification and Regression Trees methodology used by Wiegel et al.^7^, may also be a cause of such a difference.

Women with FSD in our study reported significantly low scores in all subscales of FSFI compared with women without FSD, with the lowest scores in four domains, which are desire followed by orgasm, arousal, and satisfaction domains. The difference in the remaining two domains, pain, and lubrication between the two groups is not marked and just statistically significant. This may reflect the need to give a different weight in each domain of the 6 in FSD. Desire, orgasm, and arousal may have the upper hand in the total score than pain and lubrication. Our explanation is supported by Jiann et al., who stated that desire, arousal, orgasm, and satisfaction have a direct relationship and substantial impact on female sexual function. In contrast, women's lubrication problems and sexual pain are related predominantly to biological factors^15^.

Recent recognition of the high prevalence of FSD in our society, together with the extensive investment of the pharmaceutical industry in this field along with the wide range of total FSFI score denoting FSD (2–28.1), was our main incentive to find a reliable new grading system for FSD, as women with total FSFI score two shouldn't be treated in the same way as women with a score of 28.0. Moreover, it is worth mentioning that the grading system of male erectile dysfunction invented by Rosen et al. (IIEF-5) was validated by comparing their suggested grading system with the frequency of penetration for sexual intercourse that was selected as the proxy measure for the severity of ED^16^. However, in women, there is no single domain that can solely represent sexual function.

As explained in the methodology section, our proposed new grading system classifies women (concerning their sexual function) into five categories based on FSFI total scores; severe (2–7.2), moderate (7.3–14.4), mild to moderate (14.5–21.6), mild (21.7- 28.1) cut-off value, and no FSD (28.2 -36). It depends on the answers to each question of FSFI. As each question has an ascending response score from 0/1 to 5 where 1 represents the lowest response score reflecting severe affection and 5 represents the highest response score reflecting no affection, we concluded that the sum of the scores of all “1” answers would give the patient a total score reflecting severe FSD, and that of all “2” answers will give the patient a total score reflecting moderate FSD and so forth.

When we applied this new grading system, we found that 20.4% of the studied women classified to have mild dysfunction, minimal and simple treatment may be needed in this group, this will not be the same in those who classified to have severe dysfunction (22.7) as this group may need intensified management. Therefore, we can say that our grading system seems to be simple and covers the full range of possible grades of severity and suitable for use in the clinical practice and research field. It is a critical first step in developing treatment guidelines for FSD. Such a method for reporting therapy outcomes may help to increase reporting, and thereby improve understanding of the problem and its response to different therapeutic modalities.

Furthermore, statistically significant differences between the four grades of our new grading system were reported in four domains of the 6 (table 5), which are desire, arousal, orgasm, and satisfaction, as the mean score of each domain decreased significantly, moving from mild to severe grade, which was not reported in pain and lubrication domains. This may point to the value of our new grading system in classifying FSD according to severity.

## Limitation

We were unable to compare our grading system with others because to the best of our knowledge, this is the first proposed grading system for FSD. Further research work is underway on the validity of our proposed severity classification for FSD. It includes evaluation of the degree of agreement and magnitude of the correlation between women's self-assessment of sexual function and the FSFI concerning levels of severity. It also includes studying the relationship between FSD severity and women's stress level.

## Conclusion

The reported high prevalence of FSD indicates that the need for accurate diagnoses is more significant than ever. Our new grading system intended to complement the FSFI and seems to be particularly useful in diagnosing variable degrees of FSD severity and may allow for better informed clinical decision-making.
